# Identification of Immunodominant HIV-1 Epitopes Presented by HLA-C*12:02, a Protective Allele, Using an Immunopeptidomics Approach

**DOI:** 10.1128/JVI.00634-19

**Published:** 2019-08-13

**Authors:** Takayuki Chikata, Wayne Paes, Tomohiro Akahoshi, Thomas Partridge, Hayato Murakoshi, Hiroyuki Gatanaga, Nicola Ternette, Shinichi Oka, Persephone Borrow, Masafumi Takiguchi

**Affiliations:** a Center for AIDS Research, Kumamoto University, Kumamoto, Japan; b Joint Research Center for Human Retrovirus Infection, Kumamoto University, Kumamoto, Japan; c Nuffield Department of Clinical Medicine, University of Oxford, Oxford, United Kingdom; d AIDS Clinical Center, National Center for Global Health and Medicine, Tokyo, Japan; e Jenner Institute, University of Oxford, Oxford, United Kingdom; Emory University

**Keywords:** CTL, HIV-1, HLA-C, LC-MS/MS, epitope, mass spectrometry, peptide

## Abstract

Despite the fact that the cell surface expression level of HLA-C on both uninfected and HIV-infected cells is lower than those of HLA-A and -B, increasing evidence suggests an important role for HLA-C and HLA-C-restricted CD8^+^ T cell responses in determining the efficiency of viral control in HIV-1-infected individuals. Nonetheless, HLA-C-restricted T cell responses are much less well studied than HLA-A/B-restricted ones, and relatively few optimal HIV-1 CD8^+^ T cell epitopes restricted by HLA-C alleles have been defined. Recent improvements in the sensitivity of mass spectrometry (MS)-based approaches for profiling the immunopeptidome present an opportunity for epitope discovery on a large scale. Here, we employed an MS-based immunopeptidomic strategy to characterize HIV-1 peptides presented by a protective allele, HLA-C*12:02. We identified a total of 10,799 unique 8- to 12-mer peptides, including 15 HIV-1 peptides. The latter included 2 previously reported immunodominant HIV-1 epitopes, and analysis of T cell responses to the other HIV-1 peptides detected revealed an additional immunodominant epitope. These findings illustrate the utility of MS-based approaches for epitope definition and emphasize the capacity of HLA-C to present immunodominant T cell epitopes in HIV-infected individuals, indicating the importance of further evaluation of HLA-C-restricted responses to identify novel targets for HIV-1 prophylactic and therapeutic strategies.

**IMPORTANCE** Mass spectrometry (MS)-based approaches are increasingly being employed for large-scale identification of HLA-bound peptides derived from pathogens, but only very limited profiling of the HIV-1 immunopeptidome has been conducted to date. Notably, a growing body of evidence has recently begun to indicate a protective role for HLA-C in HIV-1 infection, which may suggest that despite the fact that levels of HLA-C expression on both uninfected and HIV-1-infected cells are lower than those of HLA-A/B, HLA-C still presents epitopes to CD8^+^ T cells effectively. To explore this, we analyzed HLA-C*12:02-restricted HIV-1 peptides presented on HIV-1-infected cells expressing only HLA-C*12:02 (a protective allele) using liquid chromatography-tandem MS (LC-MS/MS). We identified a number of novel HLA-C*12:02-bound HIV-1 peptides and showed that although the majority of them did not elicit T cell responses during natural infection in a Japanese cohort, they included three immunodominant epitopes, emphasizing the contribution of HLA-C to epitope presentation on HIV-infected cells.

## Abstract (precis)

Mass spectrometry (MS)-based approaches are increasingly being employed for large-scale identification of HLA-bound peptides derived from pathogens, but only very limited profiling of the HIV-1 immunopeptidome has been conducted to date. Notably, a growing body of evidence has recently begun to indicate a protective role for HLA-C in HIV-1 infection, which may suggest that despite the fact that levels of HLA-C expression on both uninfected and HIV-1-infected cells are lower than those of HLA-A/B, HLA-C still presents epitopes to CD8^+^ T cells effectively. To explore this, we analyzed HLA-C*12:02-restricted HIV-1 peptides presented on HIV-1-infected cells expressing only HLA-C*12:02 (a protective allele) using liquid chromatography-tandem MS (LC-MS/MS). We identified a number of novel HLA-C*12:02-bound HIV-1 peptides and showed that although the majority of them did not elicit T cell responses during natural infection in a Japanese cohort, they included three immunodominant epitopes, emphasizing the contribution of HLA-C to epitope presentation on HIV-infected cells.

## INTRODUCTION

Human immunodeficiency virus type 1 (HIV-1)-specific cytotoxic T lymphocyte (CTL) responses are temporally associated with control of viremia in acute infection (1, 2), and numerous lines of evidence suggest that they play an important role in both initial containment and ongoing control of HIV-1 replication (3, 4). In preclinical models, CD8^+^ T cell-inducing prophylactic vaccine strategies have also been shown to reduce the persisting viral load established after viral challenge or even enable rapid eradication of infection in some animals (5–7). Moreover, HIV-specific CD8^+^ T cells modulate the establishment of the latent viral reservoir and contribute to the control of HIV-1 replication in tissue sites of viral persistence (8–11), providing a strong rationale for CD8^+^ T cell deployment in therapeutic strategies (10). However, HIV-1 possesses many strategies for evasion of CD8^+^ T cell control, which, together with the lack of efficacy achieved in clinical trials of CD8^+^ T cell-based prophylactic HIV-1 vaccines, such as the Step study and HVTN 505 (12, 13), and therapeutic vaccines (14), prompt a need for rational design of improved CD8^+^ T cell-inducing vaccines that elicit responses with higher efficacy (15, 16). Several studies have shown that CTLs specific for a limited number of immunodominant epitopes have the ability to control the progression of HIV-1 infection (17–21), suggesting that these epitopes would be good candidate antigens for HIV-1 vaccines. Therefore, the precise identification of these epitopes is required to inform rational vaccine design.

Identification of HLA class I-restricted HIV-1 epitopes has been performed by several strategies, including reverse-immunogenetics approaches (22–24) and screening of patient CD8^+^ T cells for responses to overlapping peptides. The former approaches are limited by their reliance on predictive algorithms trained on a limited number of data sets, where less-well-characterized alleles are generally underrepresented, and typically involve synthesis and HLA binding analysis of large numbers of peptides. In the latter approach, the overlapping peptide sets employed for response screening are often based on clade consensus sequences (20, 25, 26), meaning that responses to epitopes that differ from the clade consensus may be missed. Determination of patient autologous viral sequences and the use of autologous virus sequence-based peptides for response screening enable more comprehensive profiling of T cell responses in HIV-1-infected individuals (27–30), although the use of overlapping peptides may still result in some responses going undetected. Response-screening methods also have resource implications, being labor-intensive, entailing the synthesis of numerous peptides covering the entire HIV-1 proteome, and requiring a large number of patient peripheral blood mononuclear cells (PBMCs) for response screening.

Recent increases in the sensitivity of mass spectrometry (MS)-based approaches for epitope discovery have facilitated the identification of HLA-bound peptides on a large scale. This approach, which can be applied to cells expressing single or multiple HLA alleles, has been successfully employed to identify HLA-binding peptides derived from a number of pathogens and tumor antigens (31–35). However, only very limited profiling of the HIV-1 immunopeptidome has been conducted to date. In two studies, HLA-bound peptides were isolated from multiallelic HIV-1-infected T cell lines and primary CD4^+^ T cells, and the HLA restrictions of the peptides identified were inferred on the basis of HLA-binding motifs (36, 37), while a third study employed HIV-1-infected CD4^+^ T cells expressing soluble HLA-A*11:01 to identify HLA-A*11:01-bound HIV-1 peptides, the responses to which were then evaluated in HIV-infected individuals (38).

HLA-B alleles, which are the most polymorphic HLA class I alleles, are widely reported to be determinants of resistance/susceptibility to a variety of different diseases, including viral control and disease progression in HIV-infected individuals (39–46). In contrast to the many studies focusing on HLA-B, relatively few studies have addressed the role of HLA-C in HIV-1 infection. Cell surface expression levels of HLA-C are lower than those of HLA-B, both on uninfected cells and on cells infected with HIV, and although HLA-C expression is not downregulated by the Nef protein (47), it is downregulated by Vpu (48). This may suggest that HLA-C presents epitope peptides to CD8^+^ T cells less effectively than HLA-B.

However, a growing body of evidence has recently begun to indicate a protective role for HLA-C alleles in HIV-1 infection. A study in a cohort of 2,527 HIV-1-infected European Americans and 1,209 African Americans demonstrated a correlation between cell surface HLA-C expression levels and the efficiency of control of viremia, with subjects categorized as controllers having higher HLA-C expression levels than noncontrollers (49). Furthermore, a strong positive correlation was found between the level of HLA-C expression and HIV-specific CTL responses, suggesting that the mechanism by which increased HLA-C expression mediated protection was due at least in part to an enhanced CTL-mediated immune response. In addition, in a study in Japanese and Vietnamese HIV-infected cohorts, we demonstrated an association between HLA-C*12:02 and lower plasma viral loads and higher CD4 T cell counts (50, 51). We subsequently clarified that HLA-C*12:02-restricted CTLs (together with HLA-B*52:01-restricted T cells) effectively suppressed HIV-1 replication in chronically HIV-1-infected individuals having an HLA-B*52:01-C*12:02 haplotype (52). Together, these studies indicate that despite being expressed at lower levels than HLA-A or -B, HLA-C plays an important role in HIV-1 infection.

Notably, among the 250 optimal HIV-1 CTL epitopes defined in the LANL HIV epitope database (LANL-HSD [www.hiv.lanl.gov]), only 21 HLA-C-restricted epitopes are listed, prompting an urgent need for more comprehensive analysis of the HLA-C-bound epitope repertoire to inform prophylactic and therapeutic vaccine design. In previous studies in which we used overlapping peptides covering Gag, Pol, and Nef to screen for T cell epitopes recognized in Japanese HLA-C*12:02-positive (HLA-C*12:02^+^) subjects, we identified 4 HLA-C*12:02-restricted HIV-1 epitopes (52–54); however, it is likely that HLA-C*12:02-restricted epitopes exist in other proteins such as Env and accessory molecules.

In the present study, we employed liquid chromatography-tandem mass spectrometry (LC-MS/MS) to identify HLA-C*12:02-restricted HIV-1 peptides from HIV-1-infected cells expressing only HLA-C*12:02. In contrast to more complex MS data sets obtained from cell lines expressing multiple alleles or primary cells, the use of single-allele transfectants facilitates a more in-depth profiling of the immunopeptidome (55). We then confirmed the binding of the HIV-1 peptides identified to HLA-C*12:02 and evaluated whether T cell responses specific for these HIV-1 peptides could be detected in HIV-1-infected HLA-C*12:02^+^ individuals. Using this approach, we identified a novel HLA-C*12:02-restricted HIV-1 epitope to which immunodominant T cell responses were elicited in infected individuals. We confirmed the ability of these T cells to recognize HIV-1-infected cells and evaluated their association with clinical control of infection. Together, the results from this study enhance the understanding of the repertoire of HLA-C*12:02-binding HIV-1 and self-peptides and of HLA-C*12:02-restricted T cell responses in HIV-infected individuals.

## RESULTS

### Identification of HLA-C*12:02-restricted HIV-1 peptides by LC-MS/MS.

**FIG 1:**
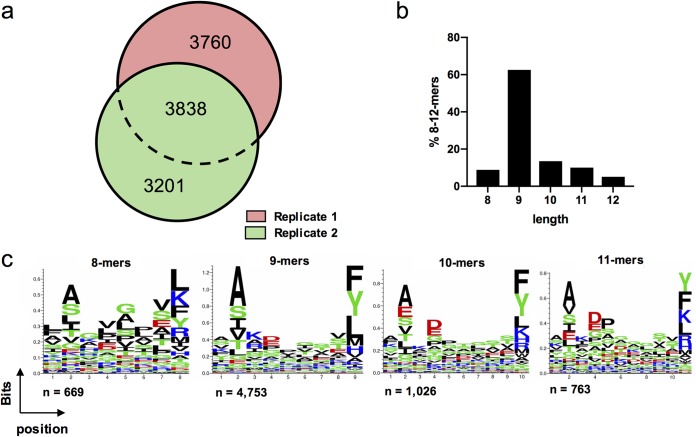
Characteristics of HLA-C*12:02-bound peptides identified by LC-MS/MS. (a) Summary of the number of unique peptides identified in HIV-1 NL4-3-infected .221-C1202 cells. The numerical overlap in peptide identification between 2 technical replicates is displayed in area-proportional Venn diagrams. (b) Length distribution of 8- to 12-mer peptides eluted from NL4-3-infected .221-C1202 cells. (c) Sequence logos of 8- to 11-mer peptides eluted from NL4-3-infected .221-C1202 cells.

721.221 cells expressing CD4 and HLA-C*12:02 (.221-C1202) were infected with the laboratory-adapted NL4-3 HIV-1 strain. HLA-peptide complexes were purified with the pan-anti-HLA class I antibody W6/32 (as described in Materials and Methods). Peptides were eluted, fractionated by high-performance liquid chromatography (HPLC), and then analyzed by LC-MS/MS. Two independent replicate experiments were performed. Background and nonspecifically coeluted peptides (56) were first removed from the two data sets, which yielded 7,598 and 7,039 C*12:02-restricted 8- to 12-mers (respectively). A total of 10,799 unique peptide sequences were identified in the two experiments (Fig. 1a). Examination of the length distribution of the 8- to 12-mer peptides identified indicated a typical HLA class I-restricted profile (Fig. 1b) (57). Analysis of the peptide motifs of 8- to 11-mers, which are the length of most of the reported CTL epitopes, revealed a preference for an alanine residue at the 2nd position (with other residues, including serine, glutamic acid, leucine, isoleucine, and valine, also being favored here) and a preference for residues including phenylalanine, tyrosine, leucine, or lysine at the C terminus in HLA-C*12:02-bound peptides (Fig. 1c).

Fifteen unique HIV-1 NL4-3-derived peptide sequences were detected across the two experiments (Table 1). Of these, Pol-IY11 and the corresponding consensus sequence of Nef-VY9 (Nef-MY9) were previously reported as immunodominant HLA-C*12:02-restricted epitopes (52, 53). The C-terminally extended Nef-VF10 peptide (VARELHPEYF) was also among the HIV-1 peptides identified. We previously demonstrated that the 9-mer version Nef-MY9 (MARELHPEY) was optimally recognized by T cells from subjects in an HIV-1-infected Japanese cohort (52).

**TABLE 1:** HIV-1 NL4-3-derived peptides eluted from HIV-1-infected HLA-C*12:02-expressing cells

Eluted NL4-3 peptide	Peptide name	Length (no. of residues)	HIV protein	Positions	Synthesized peptide (based on worldwide HIV-1 clade B)
MSQVTNPATIM	Gag-MM11	11	Gag	367–377	MSQVTNSATIM
VTNPATIM	Gag-VM8	8	Gag	370–377	VTNSATIM
FLGKIWPSH	Gag-FH9	9	Gag	433–441	FLGKIWPSH
LGKIWPSH	Gag-LH8	8	Gag	434–441	LGKIWPSH
FSVPLDKDF	Pol-FF9	9	Pol	271–279	FSVPLDKDF
ILKEPVHGVYY	Pol-IY11	11	Pol	464–474	ILKEPVHGVYY
VSAGIRKVL	Pol-VL9	9	Pol	707–715	VSAGIRKVL
VAVHVASGY	Pol-VY9	9	Pol	790–798	VAVHVASGY
DAKLVITTY	Vif-DY9	9	Vif	61–69	DAKLVITTY
MTKALGISY	Tat-MY9	9	Tat	39–47	MTKALGISY^*c*^
VTIGKIGNM	Env-VM9	9	Env	318–326	
RAIEAQQHL	Env-RL9	9	Env	557–566	RAIEAQQHL
KAAVDLSHF	Nef-KF9	9	Nef	82–90	KGALDLSHF
VARELHPEY	Nef-VY9^*a*^	9	Nef	194–202	MARELHPEY
VARELHPEYF	Nef-VF10^*b*^	10	Nef	194–203	

a Nef-VY9 was previously reported as immunodominant HLA-C*12:02-restricted epitope Nef-MY9 (52).

b Nef-VF10 included the Nef-VY9 epitope and was previously reported as Nef-MY10 (54).

c Tat-MY9 was synthesized based on the NL4-3 sequence.

### Binding of the eluted peptides to HLA-C*12:02.

During immunoprecipitation (IP) of HLA-I–peptide complexes from single HLA allele-transfected 721.221 cells, a low level of “background” peptides is nonspecifically coeluted during the IP step (56). In addition, as the parental 721.221 cell line exhibits residual expression of HLA-C*01:02 (56), peptides restricted by this allele could have been copurified with HLA-C*12:02-bound peptides. Thus, we next sought to confirm the HLA restriction of the HIV-1 peptides identified from .221-C1202 cells. The corresponding consensus sequences of the relevant NL4-3 peptides in worldwide HIV-1 clade B viruses (Table 1) were synthesized and evaluated for binding to HLA-C*12:02. As Tat-MY9 is highly polymorphic at the 1st and 2nd residues in circulating HIV-1 subtype B strains (LANL database [www.hiv.lanl.gov]), this peptide was synthesized based only on the NL4-3 sequence. Env-VM9 was not evaluated for HLA-C*12:02 binding as it was located in a highly variable region of Env, which would confound subsequent analysis of epitope recognition by T cell responses in HIV-infected HLA-C*12:02 subjects (and limit the potential utility of this epitope for targeting by vaccines). Nef-MY10 was also excluded because it included the Nef-MY9 epitope.

**FIG 2:**
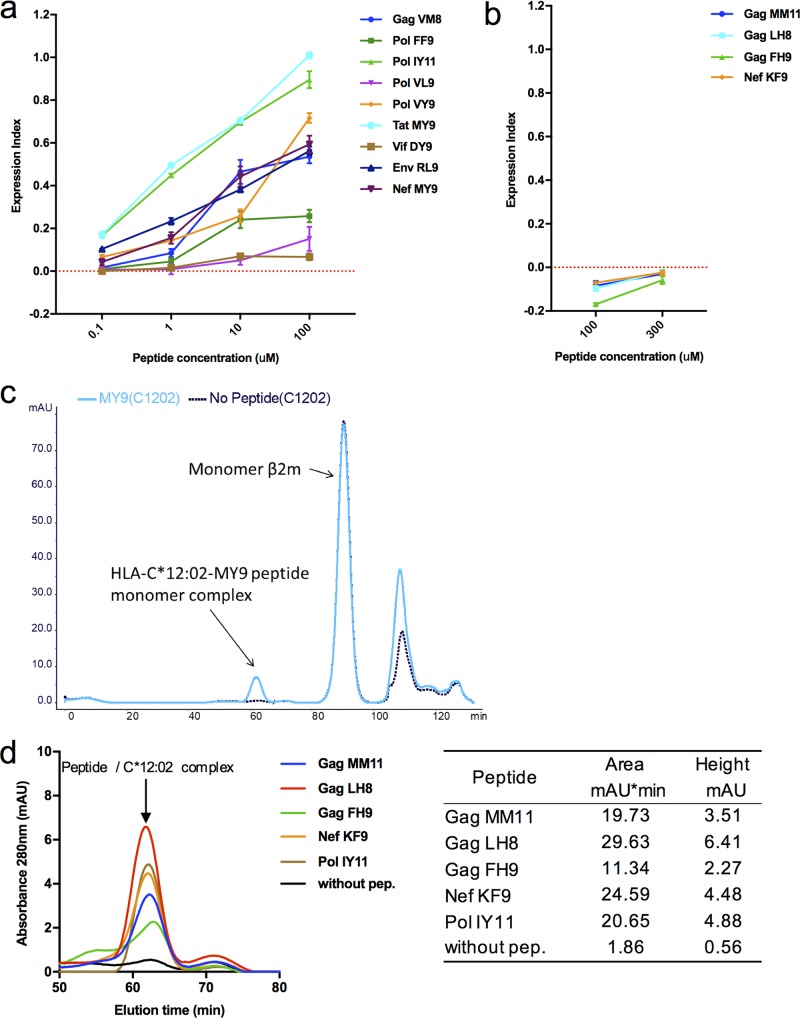
Confirmation of HLA-C*12:02 binding of HIV-1 peptides eluted from NL4-3-infected .221-C1202 cells. (a) Analysis of binding of 9 eluted peptides to HLA-C*12:02. Binding of peptides at concentrations from 0.1 to 100 μM (left) was measured by performing an HLA class I stabilization assay using RMA-S-C1202 cells. Results are plotted as the expression index, defined as the ratio of the mean fluorescence intensity (MFI) of peptide-pulsed RMA-S-C1202 cells to that of control (non-peptide-pulsed) cells kept at 26°C. (b) Analysis of binding of 4 eluted peptides to HLA-C*12:02. Binding of peptides at concentrations from 100 to 300 μM (right) was measured by performing an HLA class I stabilization assay using RMA-S-C1202 cells. Results are plotted as described above for panel a. (c) Refolding of the positive-control peptide Nef-MY9 (a known HLA-C*12:02-restricted epitope) with HLA-C*12:02 heavy chain and β_2_-microglobulin. Analysis of the products generated by fast-performance liquid chromatography shows that a complex with a predicted molecular mass coincident with the molecular mass of the complex of HLA-C*12:02, peptide, and β_2_m was generated. (d) Analysis of refolding of 4 eluted peptides that did not exhibit detectable binding to HLA-C*12:02 and Pol-IY11 (a known HLA-C*12:02-restricted epitope) in the stabilization assay.

Hence, 13 peptides were synthesized and tested for binding and stabilization of HLA-C*12:02 in an HLA stabilization assay using the Transporter 2 (TAP2)-defective RMA-S cell line expressing HLA-C*12:02 (RMA-S-C1202). It was found that 9/13 peptides (including the 2 previously reported epitopes Pol-IY11 and Nef-MY9) exhibited affinity for HLA-C*12:02 molecules, evidenced by the enhanced stabilization of surface HLA expression in a dose-dependent fashion (Fig. 2a). However, 4 peptides (Gag-MM11, Gag-LH8, Gag-FH9, and Nef-KF9) did not exhibit detectable binding to HLA-C*12:02 molecules in this assay, even when tested at a higher peptide concentration (300 μM) (Fig. 2b). To determine whether these peptides were capable of binding to HLA-C*12:02 but were of too low an affinity to enable detection of binding in the HLA stabilization assay in RMA-S cells, we measured the ability of these 4 peptides to stabilize HLA-C*12:02 using a peptide/HLA refolding assay. The refolding of the HLA monomer, β_2_-microglobulin (β_2_m), and peptide was performed under low-temperature conditions (see Materials and Methods), and the products generated were analyzed by fast protein liquid chromatography (FPLC). The refolded HLA-C*12:02 molecules formed a small peak that could be detected on the FPLC trace (data for the Nef-MY9 epitope are shown in Fig. 2c as an example). All four of the peptides tested and Pol-IY11 were shown to refold with HLA-C*12:02 heavy chain and β_2_m molecules (Fig. 2d), confirming HLA-C*12:02 restriction.

### T cell responses to HIV-1 peptides eluted from HLA-C*12:02.

**FIG 3:**
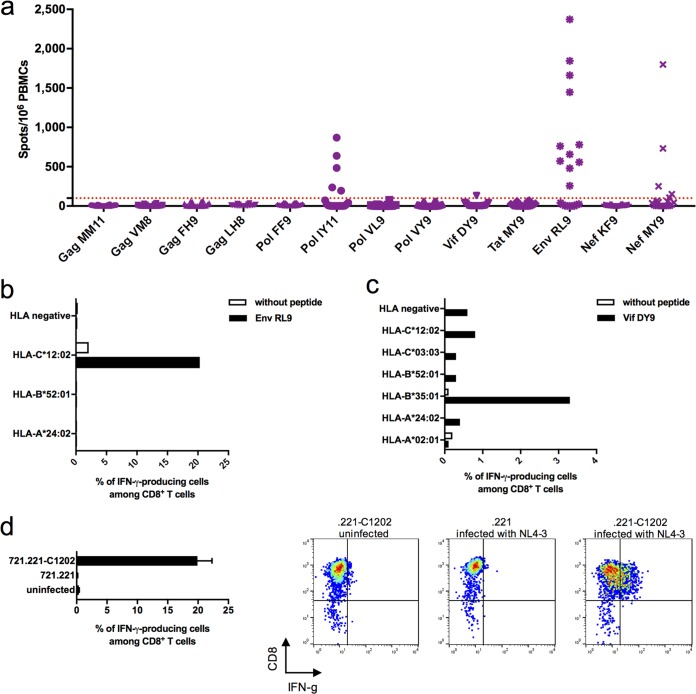
Evaluation of T cell responses to the eluted HIV-1 peptides and identification of responses to the HLA-C*12:02-restricted Env-RL9 epitope. (a) Screening for T cell responses to the eluted peptides in 20 chronically HIV-1-infected HLA-C*12:02^+^ Japanese individuals. T cell responses to 13 eluted peptides (tested at a concentration of 1 μM) were analyzed by an *ex vivo* IFN-γ ELISpot assay. A positive response was defined as >100 spots/10^6^ PBMCs. (b) Analysis of the HLA restriction of the T cell response to Env-RL9. The response of Env-RL9-expanded bulk T cells from subject KI-1407 (A*2402/–, B*5201/–, and C*1202/–) to Env-RL9 peptide-prepulsed 721.221 cell lines, each expressing a single HLA allele shared with KI-1407, was analyzed by an ICS assay. (c) Analysis of the HLA restriction of the T cell response to Vif-DY9. The response of the Vif-DY9-expanded bulk T cells from subject KI-1394 (A*0201/2402, B*3501/5201, and C*0303/1202) to Vif-DY9 peptide-prepulsed 721.221 cell lines, each expressing a single HLA allele shared with KI-1394, was analyzed by an ICS assay. (d) Recognition of NL4-3-infected cells by Env-RL9-specific CD8^+^ T cells. The response of Env-RL9-expanded bulk T cells to uninfected .221-C1202 cells and 721.221 cells and .221-C1202 cells infected with NL4-3 was analyzed by an ICS assay. Graphs at the right show representative fluorescence-activated cell sorter (FACS) data.

To explore whether these HLA-C*12:02-restricted HIV-1 peptides were epitopes to which T cell responses were elicited during natural HIV-1 infection, we screened for CD8^+^ T cell responses to these 13 peptides in 20 HIV-1-infected HLA-C*12:02^+^ individuals using *ex vivo* gamma interferon (IFN-γ) enzyme-linked immunosorbent spot (ELISpot) assays. T cell responses to 4/13 peptides were detected in one or more individuals (Fig. 3a). These included the previously described Pol-IY11 and Nef-MY9 epitopes as well as two additional C*12:02-restricted peptides (Env-RL9 and Vif-DY9). Of the 20 individuals tested, 13 exhibited T cell responses to the Env-RL9 peptide, and 1 individual showed T cell reactivity to Vif-DY9. T cell responses to Pol-IY11 and Nef-MY9 were also detected in 5/20 individuals, consistent with results obtained in previous studies in Japanese cohorts, where responses to these epitopes were observed in a similar proportion of infected individuals (52, 53).

To confirm the HLA restriction of the T cell responses to Env-RL9 and Vif-DY9, we expanded T cells specific for Env-RL9 by stimulating PBMCs from the responder KI-1407 (A*24:02/24:02, B*52:01/52:01, and C*12:02/12:02) with the Env peptide and T cells specific for Vif-DY9 by stimulating PBMCs from the responder KI-1394 (A*02:01/24:02, B*35:01/52:01, and C*03:03/12:02) with the Vif peptide. The cultured T cells were tested for their ability to recognize the peptides of interest presented on 721.221 cells expressing each of the HLA alleles possessed by subject KI-1407 or KI-1394, reading out responses by intracellular cytokine staining (ICS). The T cells recognized .221-C1202 cells prepulsed with Env-RL9 but not prepulsed .221-A2402 or .221-B5201 cells (Fig. 3b), indicating that the T cell response to Env-RL9 was restricted by HLA-C*12:02. In contrast, Vif-DY9-reactive T cells expanded from subject KI-1394 showed a higher-magnitude response to Vif-DY9-pulsed HLA-B*35:01-expressing cells than to Vif-DY9-pulsed HLA-C*12:02-expressing cells, suggesting that the dominant Vif-DY9 response in this individual was HLA-B*35:01 restricted (Fig. 3c). Indeed, Vif-DY9 was reported to be an HLA-B*35:01-restricted epitope in a previous study (58). The Vif-DY9 peptide can bind to both HLA-C*12:02 and HLA-B*35:01, as these two alleles have similar peptide-binding motifs (59). The results presented here, together with multiple other reports indicating that a given peptide sequence may be presented by more than one HLA allele (LANL database [www.hiv.lanl.gov]), demonstrate the importance of confirming that responses observed in patients to peptides eluted from one HLA molecule are indeed mediated by T cells restricted by the allele of interest.

We next investigated whether Env-RL9-specific T cells could recognize NL4-3-infected .221-C1202 cells. Env-RL9-specific T cells recognized NL4-3-infected .221-C1202 but not uninfected .221-C1202 cells (Fig. 3d), confirming that Env-RL9 was a novel HLA-C*12:02-restricted epitope effectively presented on HIV-1-infected cells. The results from *ex vivo* ELISpot assays showed that the average magnitude of the T cell responses to Env-RL9 in individuals recognizing this epitope (575 spot-forming cells [SFC]/10^6^ PBMCs) was significantly higher (*P* = 0.014 and 0.01, respectively) than that of the responses to Pol-IY11 or Nef-MY9 in individuals recognizing these epitopes (130 and 167 SFC/10^6^ PBMCs, respectively) (Fig. 3a). Taken together, these results indicate that Env-RL9 is an immunodominant HLA-C*12:02-restricted T cell epitope.

**FIG 4:**
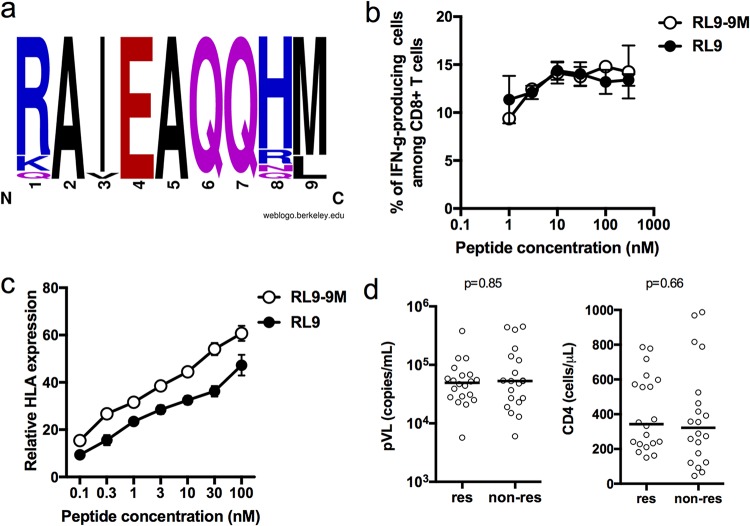
Effects of L-to-M mutation of the C-terminal residue in the Env-RL9 epitope on T cell recognition and binding affinity of mutant peptides. (a) Logo of the region of sequence containing the Env-RL9 peptide in the autologous virus of 20 chronically HIV-1-infected HLA-C*12:02^+^ Japanese individuals who were analyzed for T cell responses to this epitope. (b) Recognition of Env-RL9 and Env-RL9-9M peptides by CD8^+^ T cells. The responses of bulk T cells expanded by stimulation with Env-RL9 to .221-C1202 cells prepulsed with Env-RL9 or Env-RL9-9M peptides at concentrations from 1 to 300 nM were analyzed by an ICS assay. (c) Binding of Env-RL9 and Env-RL9-9M peptides to HLA-C*12:02. The relative binding abilities of the two peptides were measured by performing HLA class I stabilization assays with RMA-S-C1202 cells using peptide concentrations from 0.1 to 300 μM. Results are plotted as the expression index, defined as the ratio of the MFI of peptide-pulsed RMA-S-C1202 cells to that of control (non-peptide-pulsed) cells kept at 26°C. (d) T cell responses to Env-RL9 (tested at a concentration of 1 μM) were analyzed in 40 HLA-C*12:02^+^ individuals by an ELISpot assay. Subjects were defined as responders (res) if they exhibited Env-RL9 responses of >100 spots/10^6^ PBMCs. Plasma viral loads (pVL) and CD4 T cell counts of responder and nonresponder (non-res) subjects are shown. The statistical significance of differences in plasma viral loads and CD4 counts between Env-RL9 nonresponders and responders was analyzed using a Mann-Whitney test.

Analysis of the sequence of Env-RL9 in the 20 HLA-C*12:02^+^ individuals screened for T cell responses revealed that more than 80% of these individuals had M at position 9 of the epitope (Fig. 4a). We therefore analyzed T cell responses to Env-RL9-9M and Env-RL9 using T cells expanded with Env-RL9 from epitope-reactive donors. The results showed that these CD8^+^ T cells recognized both epitopes (Fig. 4b). The HLA stabilization assay also showed no remarkable difference in binding affinity between Env-RL9 and Env-RL9-9M peptides (Fig. 4c). Thus, Env-RL9-specific T cells could effectively cross-recognize Env-RL9 and Env-RL9-9M. To enable evaluation of the impact of Env-RL9-specific T cells on clinical outcome in HIV-1-infected individuals, we screened an additional 20 HIV-1-infected HLA-C*12:02^+^ individuals for the presence of responses to this epitope. Altogether, 20/40 individuals showed responses to the RL9 peptide. However, there was no difference in plasma viral loads or CD4 T cell counts between responders and nonresponders (Fig. 4d), suggesting that the presence of Env-RL9-specific responses was not a dominant determinant of viral control and disease progression in this Japanese HIV-1-infected cohort.

### HLA-C*12:02 binding and T cell recognition of HLA-C*12:02 peptide variants found in circulating HIV-1.

**FIG 5:**
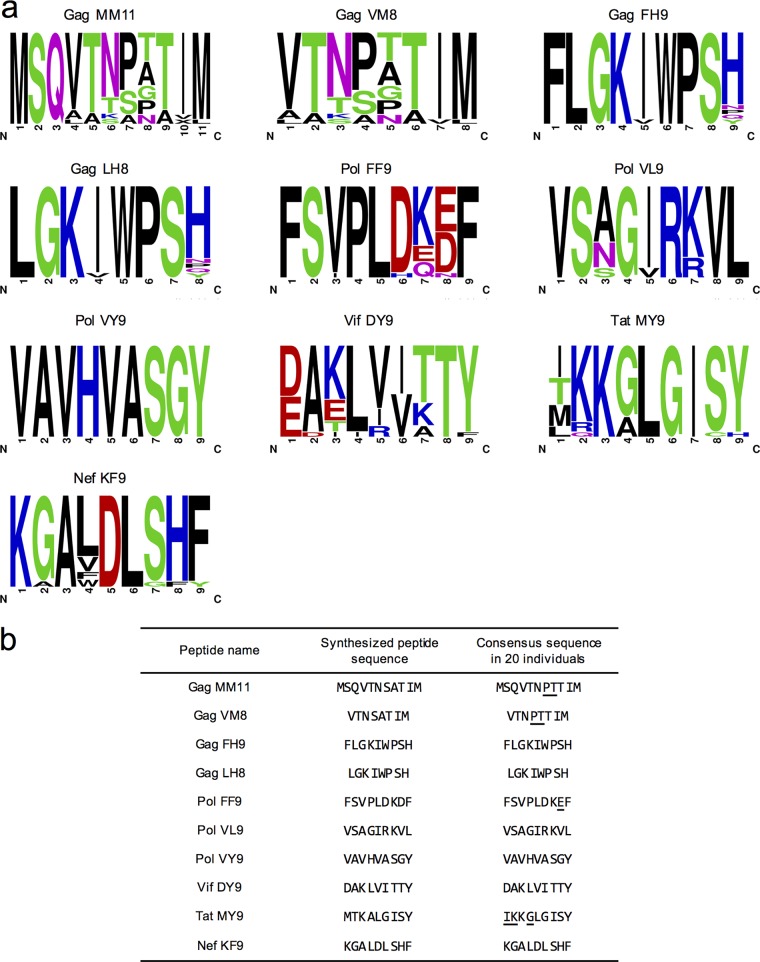
Sequence variation in Japanese subjects in HLA-C*12:02-binding HIV-1 peptides to which no T cell responses were detected in infected individuals. (a) Logos of the autologous virus sequences of 10 peptides to which no T cell responses were observed in 20 chronically HIV-1-infected HLA-C*12:02^+^ Japanese individuals in whom T cell response screening was performed. (b) List of sequences of peptides chosen for synthesis based on the worldwide clade B consensus sequence in the LANL database and the consensus sequence in the 20 chronically HIV-1-infected HLA-C*12:02^+^ Japanese individuals evaluated here. Amino acid residues that differ in the worldwide subtype B consensus sequence and the consensus sequence in the 20 infected individuals studied here are underlined.

**FIG 6:**
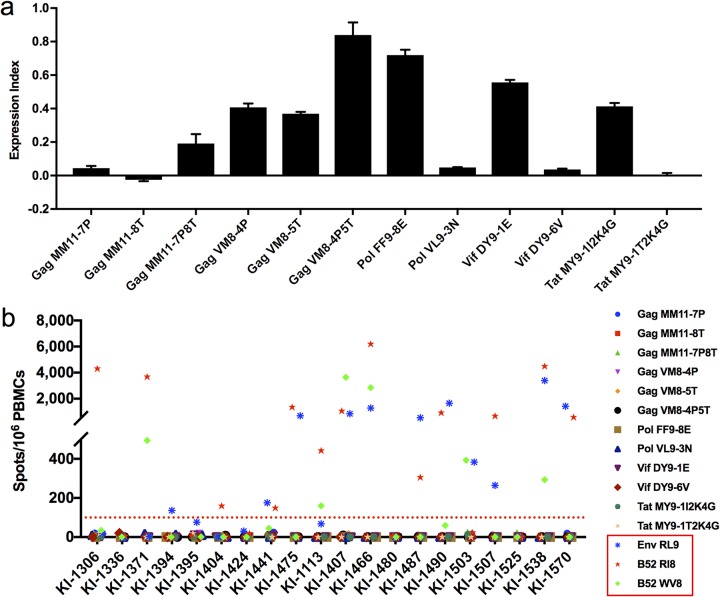
HLA-C*12:02 binding of four 20-patient consensus sequences and eight additional variant peptides and T cell responses to these peptides. (a) Binding of four 20-patient consensus sequences and eight additional variant peptides to HLA-C*12:02. Peptide binding was evaluated using 100 μM peptide in an HLA class I stabilization assay with RMA-S-C1202 cells. Results are plotted as the expression index, defined as the ratio of the MFI of peptide-pulsed RMA-S-C1202 cells to that of control (non-peptide-pulsed) cells kept at 26°C. (b) T cell responses to four 20-patient consensus and eight additional variant peptides in 20 chronically HIV-1-infected HLA-C*12:02^+^ Japanese individuals. Responses to the peptides indicated (tested at a concentration of 1 μM) were analyzed by an IFN-γ ELISpot assay. The peptides boxed in red are 3 positive-control peptides (Gag-RI8, Gag-WV8, and Env-RL9). A positive response was defined as >100 SFC/10^6^ PBMCs.

Having found no evidence of HLA-C*12:02-restricted T cell recognition of 10 of the 13 peptides shown to be presented with HLA-C*12:02 in cells infected with HIV-1 NL4-3, we sought to determine the impact that differences within these epitope sequences between the NL4-3/worldwide clade B viruses and those present in the subjects screened for T cell reactivity may have had on the detection of responses to these peptides. We first determined the amino acid sequences of the 10 peptides (9 nonrecognized peptides plus Vif-DY9) in the 20 HLA-C*12:02^+^ individuals in whom peptide-specific responses had been evaluated (Fig. 5a). The 20-subject consensus sequences of 6 of the peptides matched the worldwide subtype B consensus sequence (Fig. 5b). In contrast, for Gag-MM11, Gag-VM8, Pol-FF9, and Tat-MY9, we found that their 20-subject consensus sequences (Gag-MM11-7P8T, Gag-VM8-4P5T, Pol-FF9-8E, and Tat-MY9-1I2K4G) were different from those of the previously evaluated Japanese population subtype B consensus sequence peptides (Fig. 5b). After further analysis of amino acid substitutions in the patient autologous viruses used to form the whole-group consensus sequence, we selected 8 additional peptides with substitutions observed in more than 5 individuals (Fig. 6a). We synthesized these 4 patient-consensus and 8 additional variant peptides and tested their binding to the HLA-C*12:02 molecule at a concentration of 100 μM by an HLA stabilization assay (Fig. 6a). These peptides all showed measurable affinities for the HLA-C*12:02 molecule with the exception of Gag-MM11-8T and Tat-MY9-1T2K4G. Therefore, we went on to investigate T cell responses to these peptides in the 20 HIV-1-infected HLA-C*12:02^+^ individuals in whom responses to the NL4-3/worldwide subtype B consensus sequence peptides had previously been tested by an IFN-γ ELISpot assay. Since all 20 of these HLA-C*12:02^+^ individuals were also HLA-B*52:01^+^, we also tested for responses to two previously reported HLA-B*52:01-restricted epitopes (Gag-RI8 and Gag-WV8) (53) as well as the HLA-C*12:02-restricted Env-RL9 peptide in parallel (as positive controls). T cell responses to the positive-control peptides Env-RL9, Gag-RI8, and/or Gag-WV8 were observed in 16/20 of the subjects tested, but no T cell responses to the 12 test HLA-C*12:02 peptides were detected in these individuals (Fig. 6b). These results suggested that these peptides were not T cell epitopes, although we cannot exclude the possibility that they were subdominant epitopes, T cell responses to which were present in a minority (<5%) of HIV-1-infected HLA-C*12:02^+^ individuals.

## DISCUSSION

Recent advances in the sensitivity of LC-MS/MS-based approaches for characterizing cellular immunopeptidomes have provided an opportunity for large-scale discovery of tumor- and pathogen-derived HLA class I-binding peptides. In the current study, we profiled the repertoire of peptides eluted from HIV-1-infected cells expressing a single HLA-C allele, HLA-C*12:02, identifying 15 unique HIV-1-derived peptides, including 2 peptides previously shown to comprise HLA-C*12:02-restricted epitopes recognized by immunodominant CTL responses in HIV-infected individuals (52, 53) and 13 peptides not previously confirmed as HLA-C*12:02-restricted epitopes. Two additional HLA-C*12:02-restricted HIV-1 epitopes, Gag-TH9 and Pol-KY9, to which subdominant T cell responses were observed in previous studies in Japanese HIV-infected individuals (52, 54), were not detected in the current study, despite the use of cells overexpressing HLA-C*12:02. This may reflect the limitations of current LC-MS/MS-based epitope discovery workflows, where less-abundant peptides within complex samples may not be reproducibly detected (60, 61). Another caveat of the approach used here for peptide identification is that lower-affinity peptides are underrepresented in the data sets obtained, as they may dissociate from HLA-I during the immunoprecipitation step (56). Nonetheless, LC-MS/MS-based immunopeptidome profiling still provides a useful method for identification of novel epitopes restricted by HLA alleles which are not well characterized. Moreover, by profiling all the self-peptides and HIV peptides presented on a single HLA-expressing cell line, we were able to correctly define the HLA-C*12:02 peptide-binding motif, which will facilitate more accurate prediction of putative HLA-C*12:02-binding peptides in future studies than would be possible using the version of the HLA-C*12:02 motif currently reported in the major histocompatibility complex (MHC) motif viewer (59).

No T cell responses were observed to 10 of the confirmed HLA-C*12:02-binding NL4-3 peptides (or variant versions thereof present in our Japanese HIV-infected subjects) in 20 HLA-C*12:02^+^ HIV-infected individuals, suggesting that these peptides do not efficiently elicit robust priming of epitope-specific T cell responses during natural HIV-1 infection. In a recent study using the same methodology as the one in the present study, we identified 14 HLA-A*11:01-binding HIV-1 peptides, 43% of which were recognized by T cell responses in infected individuals (our unpublished observation), a value almost double that of the 23% of HLA-C*12:02-restricted peptides tested here to which responses were detected. Although further studies with additional HLA-A, HLA-B, and HLA-C alleles will be required to enable robust conclusions to be drawn about differences in the efficiency with which epitopes presented by HLA-A/B and HLA-C alleles induce CD8^+^ T cell priming in HIV-infected individuals, it is tempting to speculate that the lower level of expression of HLA-C than of HLA-A and -B molecules on the surface of both uninfected (62, 63) and HIV-1-infected (48) cells may result in less effective induction of CD8^+^ T cell responses to HLA-C than HLA-A and -B epitopes.

HLA-bound peptide complexes are recognized not only by T cell receptors but also by killer cell immunoglobulin-like receptors (KIRs) (64). KIR2DL/DS can recognize HLA-C1 group alleles, including HLA-C*12:02. Following the engagement of KIR2DS with a cognate HLA-peptide complex, NK cells can be activated via ITAM-based pathways to release the contents of their lytic granules toward target cells, whereas this activity of NK cells is inhibited via ITIM-based signaling pathways following recognition of HLA-peptide complexes by KIR2DL (64–66). Some of the HLA-C*12:02-binding HIV-1 peptides that were not recognized by HLA-C*12:02-restricted T cells might serve as ligands for KIR that bind HLA-C*12:02 and modulate immune responses in infected individuals via that mechanism. Indeed, a previous study showed that HLA-C*12:02− and HLA-C*14:03−HIV-1 peptide complexes act as ligands of KIR2DL (67). Future analysis of KIR binding to complexes of HLA-C*12:02 bound to the peptides to which no T cell responses were detected in infected individuals should clarify the role of these peptides in immune recognition by NK cells and KIR-expressing CD8^+^ T cells.

In addition to Nef-VY9, which was previously reported as immunodominant HLA-C*12:02-restricted epitope MY9 (52, 53), the C-terminally extended Nef-VF10 peptide (VARELHPEYF) was also among the HIV-1 peptides identified here. The consensus version of this epitope in worldwide subtype B virus sequences reported in the LANL database is MARELHPEYY. In a previous study, we showed that PBMCs expanded by *in vitro* culture with a cocktail of peptides, including MARELHPEYY, recognized MARELHPEY (Nef-MY9) much more efficiently than MARELHPEYY (Nef-MY10) (52), despite the fact that the affinities of binding of these peptides to HLA-C*12:02 were similar (data not shown). We cannot preclude that some T cell clones may recognize Nef-MY10 better than Nef-MY9, but these results suggest that Nef-MY9 is the optimal-length epitope for recognition by the majority of T cells. However, Nef-MY9-specific T cells may cross-recognize the Nef-MY10 peptide with lower avidity, and/or, as discussed above, the Nef-MY10 peptide bound to HLA-C*12:02 might constitute a good ligand for KIR recognition.

Env-RL9 is located at heptad repeat 1 (HR1) of gp41, which is a comparatively conserved sequence, suggesting that Env-RL9 might be presented in the majority of HIV-infected individuals. Indeed, in our cohort, 50% of subjects tested exhibited a detectable response to this epitope. Although the most common amino acid at the C terminus of the epitope sequence in viruses from patients in our cohort differed from that present in NL4-3, the Env-RL9-9M peptide variant showed a similar affinity of binding to HLA-C*12:02 to that of Env-RL9 and was also recognized by T cells. These results indicated that both Env-RL9 and RL9-9M are cross-recognized by epitope-reactive T cells. However, subjects exhibiting responses to Env-RL9 had persisting viral loads and CD4 T cell counts that did not differ from those in subjects without epitope-specific responses, indicating a lack of a correlation of responses to this epitope with clinical outcomes in this cohort. Previous studies have also shown that T cell responses to epitopes in the Env region are not associated with good clinical outcomes (68–70) and are positively correlated with the plasma viral load in Africans (18). These findings suggest that the majority of Env-specific CTL responses do not mediate effective sustained suppression of HIV-1. This may be due in part to the high sequence variation that can be tolerated in Env, which enables the virus to escape from Env-reactive T cell responses with relatively low costs to intrinsic viral fitness.

In summary, the present study highlights the utility of LC-MS/MS-based approaches for the identification of HLA-bound viral peptides expressed on HIV-1-infected cells. Three immunodominant epitopes, including two previously reported epitopes, were detected among the peptides eluted from HLA-C*12:02 single-allele-transfected cells. Unlike responses to the other immunodominant HLA-C*12:02-restricted epitopes, Env-RL9-specific responses were not found to correlate with protection in our cohort, suggesting that T cell responses to the Pol-IY11 and Nef-MY9 epitopes (both of which are positively associated with a good clinical outcome in HIV-1-infected Japanese individuals [52, 53]) play a more important role in *in vivo* control of viral replication. Although the majority of the peptides presented by HLA-C*12:02 were not observed to have elicited T cell responses during natural infection in our cohort, the previously documented ability of HLA-C-restricted CD8^+^ T cells to influence HIV-1 control suggests the potential utility of vaccine-mediated induction of T cell responses to these novel HLA-C-restricted viral epitopes to enhance the efficacy of prophylactic and/or therapeutic HIV-1 control. Furthermore, the identification of T cell epitopes by use of this approach could be useful in future studies of other infectious diseases and cancers.

## MATERIALS AND METHODS

### Ethics statement.

All treatment-naive Japanese adult individuals chronically infected with HIV-1 subtype B were recruited from the National Center for Global Health and Medicine. The study was approved by the ethics committees of Kumamoto University (RINRI-1340 and GENOME-342) and the National Center for Global Health and Medicine (NCGM-A-000172-01). Written informed consent was obtained from all individuals for the collection of blood and their subsequent analysis according to the Declaration of Helsinki.

### HLA genotyping.

HLA genotypes of the HLA-A, -B, and -C alleles were identified by the Luminex microbead method at the HLA laboratory (Japan).

### Cell lines.

The HLA class Ia-deficient 721.221 cell line expressing CD4 and transfected with HLA-C*12:02 (.221-C1202) was generated in a previous study (54). The TAP2-deficient mouse RMA-S cell line expressing HLA-C*12:02 (RMA-S-C1202) was generated as previously described (71). Both cell lines were cultured in RPMI 1640 medium (Thermo Fisher) containing 10% fetal calf serum (FCS) and 0.15 mg/ml hygromycin B (Calbiochem).

### HIV-1 NL4-3 infection.

.221-C1202 cells were infected with HIV-1 NL4-3 in a low volume of RPMI 1640 medium (Thermo Fisher) containing 10% fetal bovine serum (FBS), 2 mM l-glutamine, 100 U/ml penicillin, 100 μg/ml streptomycin, and 10 mM HEPES (R10). Fifty microliters of NL4-3 stock virus (reverse transcriptase [RT] value = 4 × 10^2^ ng/ml) was added to 8 × 10^6^ .221-C1202 cells, and the cells were then incubated for 1.5 h at 37°C, after which 20 ml of R10 was added and the cells were cultured overnight. At day 1 postinfection, a further 20 ml R10 was added, and cells were split (1:2) on day 2. On day 3 postinfection, the proportion of cells infected with HIV-1 was determined by intracellular p24 staining, as previously described (72). Infected flasks were then harvested for immunoprecipitation of HLA-peptide complexes.

### HLA class I immunoprecipitation.

A total of 1.5 × 10^8^ to 2 × 10^8^ .221-C1202 cells infected with NL4-3 were harvested, washed in phosphate-buffered saline (PBS), and lysed in 5 ml of lysis buffer (1% IGEPAL 630, 300 mM NaCl, and 100 mM Tris [pH 8.0] plus protease inhibitors) at 4°C for 45 min. Two centrifugation steps (2,000 × *g* for 10 min followed by 20,000 × *g* for 30 min at 4°C) were next employed to clear the lysates of infected cells prior to overnight capture of HLA-peptide complexes on W6/32-coated protein A‐Sepharose beads. W6/32-bound HLA-peptide complexes were sequentially washed with 10 to 20 ml of wash buffer 1 (0.005% IGEPAL, 50 mM Tris [pH 8.0], 150 mM NaCl, 5 mM EDTA), wash buffer 2 (50 mM Tris [pH 8.0], 150 mM NaCl), wash buffer 3 (50 mM Tris [pH 8.0], 400 mM NaCl), and, finally, wash buffer 4 (50 mM Tris [pH 8.0]) under gravity flow in Econo-Column glass chromatography columns (Bio-Rad). Peptide-HLA complexes were eluted from the beads with 5 ml of 10% acetic acid, and following drying under a vacuum, they were loaded onto a 4.6- by 50-mm ProSwift RP-1S column (Thermo Fisher Scientific) and eluted from an Ultimate 3000 HPLC system (Thermo Scientific) using a 500-μl/min flow rate over 10 min from 2 to 34% buffer B (0.1% trifluoroacetic acid [TFA] in acetonitrile) in buffer A (0.1% TFA in water). Alternate 1-ml fractions (odd and even) were divided into two separate pools and dried under vacuum prior to resuspension in loading buffer for LC-MS/MS analysis.

### Liquid chromatography-tandem mass spectrometry.

Each HPLC-eluted sample was resuspended in 20 μl loading buffer, and 9 μl of it was injected onto a 3-μm-particle-size, 0.075-mm by 150-mm Acclaim PepMap rapid separation liquid chromatography (RSLC) C_18_ column and further loaded onto a 2-μm-particle-size, 75-μm by 50-cm Acclaim PepMap RSLC C_18_ column using an Ultimate 3000 nanoflow ultrahigh-performance liquid chromatography (nUPLC) system (Thermo Scientific). A linear gradient of 3 to 25% buffer B (0.1% formic acid and 5% dimethyl sulfoxide [DMSO] in acetonitrile) in buffer A (0.1% formic acid and 5% DMSO in water) was applied over 1 h to elute peptides from the column (flow rate of 250 μl/min). Peptides were introduced, using an Easy-Spray source at 2,000 V at 40°C, to a Fusion Lumos mass spectrometer (Thermo Scientific). The ion transfer tube temperature was set to 305°C. Full MS spectra were recorded from *m/z* 300 to 1,500 in an Orbitrap instrument at a resolution of 120,000 with an automatic gain control (AGC) target of 400,000. Precursors were selected in top-speed mode within a 2-s cycle time (accumulation time of 120 ms) and an isolation width of 1.2 atomic mass units (amu) for fragmentation. Higher-energy collisional dissociation (HCD) with a collision energy setting of 28 was performed on the peptides with a charge state of 2 to 4, while a higher collision energy of 32 was applied to singly charged precursor ions that were selected with lower priority. MS resolution was set at 120,000, and MS2 resolution was set at 30,000. All fragmented precursor ions were actively excluded from repeated selection for 30 s.

### Analysis of LC-MS/MS data sets.

The analysis of all LC-MS/MS data sets (.raw files) was performed using PEAKS v8.0 software (Bioinformatic Solutions). No enzyme was specified during the peptide spectral matching, and mass tolerance settings of 5 ppm (for precursor ions) and 0.03 Da (for fragment ions) were used. Spectral sequence annotation was performed against the annotated Homo sapiens Swiss-Prot database appended with a 6-frame translation of the HIV-1 NL4-3 genome. A false discovery rate of 5% was set using a parallel decoy database search. Shannon sequence logos for all unique database-matched (Homo sapiens and HIV) 8-mer to 12-mer peptides were produced using the online tool Seq2logo v2.0 (73).

### HLA stabilization assay.

The affinity of peptide binding to HLA-C*12:02 was examined using RMA-S-C1202 cells as previously described (67). Briefly, RMA-S-C1202 cells were cultured at 26°C for 16 h and then pulsed with peptides at 26°C for 1 h and subsequently incubated at 37°C for 3 h. Staining of cell surface HLA-C molecules was performed with DT-9, an HLA-C-specific monoclonal antibody (mAb) (a gift from Mary Carrington, NIH), and fluorescein isothiocyanate (FITC)-conjugated sheep anti-mouse IgG (Jackson ImmunoResearch). Staining data were acquired on a FACSCanto II instrument (BD Biosciences) and analyzed using FlowJo 10.5.3 software. Relative HLA expression was calculated as the ratio of the mean fluorescence intensity (MFI) of peptide-pulsed RMA-S-C1202 cells to that of control (non-peptide-pulsed) cells kept at 26°C.

### Refolding assay.

Refolding of peptides with the HLA heavy chain and β_2_m was performed as previously described (74). Briefly, HLA-C*12:02 heavy chain and β_2_m were overexpressed in Escherichia coli as inclusion bodies and solubilized with 8 M urea. Before refolding, only β_2_m was refolded and purified with Superdex 75 pg 26/600 (GE Healthcare) chromatography, and the solubilized HLA heavy chain was treated with 6 M guanidine hydrochloride at 4°C overnight. The dissolved HLA heavy chain, purified monomer β_2_m, and peptide (final concentrations of 20 μM, 2 μM, and 85 μM, respectively) were diluted in 10 ml of refolding buffer (100 mM Tris-HCl [pH 8.0], 400 mM l-arginine, 2 mM EDTA-Na, 5 mM reduced l-glutathione, and 0.5 mM oxidized l-glutathione). After 48 h of slow stirring at 4°C, the peptide–HLA-C complex was then concentrated and purified by Superdex 75 pg 26/600 (GE Healthcare) chromatography.

### ELISpot assay.

Peripheral blood mononuclear cells (PBMCs) were separated from whole blood and stored at −80°C. ELISpot assays were performed as previously described (52). Briefly, 1 × 10^5^ PBMCs from HIV-1-infected individuals and 100 nM each C*12:02-restricted HIV-1 peptide were added to 96-well polyvinylidene plates (Millipore) that had been coated overnight with 5 μg/ml anti-IFN-γ mAb 1-D1K (Mabtech). The plates were then incubated for 16 h at 37°C and subsequently washed with PBS before the addition of biotinylated anti-IFN-γ mAb (Mabtech) at 1 μg/ml. After the plates had been incubated at room temperature for 90 min, they were washed with PBS and then incubated with streptavidin-conjugated alkaline phosphatase (Mabtech) for 60 min at room temperature. Following washes with PBS, individual cytokine-producing cells were visualized as dark spots after a 20-min reaction with 5-bromo-4-chloro-3-indolyl phosphate and nitroblue tetrazolium in the presence of an alkaline phosphatase-conjugated substrate (Bio-Rad). The spots were counted with an Eliphoto-Counter (Minerva Teck). The number of spots was calculated per 10^6^ PBMCs. One hundred spots per 10^6^ PBMCs were defined as a positive response, as described previously (52).

### Intracellular cytokine staining assay.

PBMCs from HIV-1-infected individuals were expanded by culture with each HIV-1 peptide for 2 weeks. .221-C1202 cells prepulsed with each HIV-1 peptide or .221-C1202 cells infected with HIV-1 NL4-3 were added to a 96-well plate together with bulk-cultured T cells, and the cells were incubated for 4 h at 37°C with brefeldin A (10 μg/ml). The cells were then stained with peridinin chlorophyll protein (PerCP)-labeled anti-CD3 mAb (BioLegend), phycoerythrin (PE)-labeled anti-CD4 mAb (BioLegend), and allophycocyanin (APC)-labeled anti-CD8 mAb (Dako) and subsequently fixed with 4% paraformaldehyde and incubated in permeabilization buffer (0.1% saponin–10% FBS–PBS). Thereafter, the cells were stained with FITC-labeled anti-IFN-γ mAb (BioLegend). Staining data were acquired on a FACSCanto II instrument (BD Biosciences) and analyzed using FlowJo 10.5.3 software.

### Bulk HIV-1 sequencing.

Plasma was separated from whole blood and stored at −80°C. Bulk sequencing of plasma viral RNA from 20 HIV-1-infected patients was performed as described previously (75). Sequence logos were generated by using WebLogo, version 2.8.2 (76).

### Statistical analyses.

Unless otherwise stated, the statistical significance of differences between groups was calculated by using the Mann-Whitney U test in GraphPad Prism v7.0. Differences were considered statistically significant at a *P* value of <0.05.
